# High myopia and macular vascular density: an optical coherence tomography angiography study

**DOI:** 10.1186/s12886-021-02156-2

**Published:** 2021-11-27

**Authors:** Yang Jiang, Shang Lou, Ying Li, Youxin Chen, Thomas Chengxuan Lu

**Affiliations:** 1 Department of Ophthalmology, Peking Union Medical College Hospital, Chinese Academy of Medical Sciences, Beijing, 100730 China; 2 Key Laboratory of Ocular Fundus Diseases, Chinese Academy of Medical Sciences & Peking Union Medical College, Beijing, China; 3 Department of Ophthalmology, Jiangxi Cancer Hospital, Nanchang Beijing ease road No.519, Jiang xi, China; 4 School of Medicine, University of New South Wales, Kensington, NSW 2052 Australia

## Abstract

**Objective:**

To investigate whether there are differences inmacular vascular density (VD) between patients with high-myopia (HM) and those with non-high myopia (NHM) using Optical Coherence Tomography Angiography (OCTA).

**Method:**

OCTA was performed on 35 eyes with HM with spherical equivalence (SE) > − 6.00D and 35 eyes with NHM with SE ≤ -6.00D. Vascular densities of the macula (overall macula, fovea, parafovea, superior hemi and inferior hemi) were measured in each of the superficial, deep and choriocapillaris layers of the retina.

**Results:**

In the superficial retinal layer, overall macular VFD was significantly higher in the NHM compared to the HM group (51.27 ± 3.74 vs. 48.07 ± 5.69, *p* < 0.05). There were significant differences between the NHM and HM in parafovea (52.58 ± 5.78 vs. 49.4 ± 6.43, *p* < 0.05), superior-hemi (53.38 ± 4.03 vs 49.78 ± 6.84, *p* < 0.05) and inferior-hemi regions (53.49 ± 4.61 vs 49.05 ± 6.41, *p* < 0.05), but not in the fovea region. Similarly, in the deep retinal layer, overall macular VFD was significantly higher in the NHM group compared to the HM group (58.69 ± 2.46 vs. 56.90 ± 4.08, *p* < 0.05). There was significant differences between the HM and NHM in superior-hemi region (61.97 ± 2.68 vs. 60.08 ± 3.98, *p* < 0.05), but not in the fovea, parafovea, and inferior-hemi region. In the choriocapillaris, there was no difference in the overall macular VFD, nor any of the individual sectors between the HM and the NHM groups.

**Conclusion:**

VFD in the superficial and deep retinal layers of the macula are significantly increased in the NHM compared to HM eyes. This is not the case in the choroidal capillary layers of the retina.

## Background

Uncorrected refractive error is common, affecting approximately 108 million people worldwide. It is also the second leading cause of blindness worldwide [1]. In a Japanese population study, 12.2% of vision impairment was caused by myopic macular degeneration [2]. Among other population-based studies, the prevalence of high myopia (HM) has been estimated to comprise 5 to 10% of diagnosed myopias, and 1 to 4% of the general population [3, 4]. In the US population, it is estimated that HM has increased 8-fold over 30 years, from 0.2 to 1.6% [5]. Myopia therefore poses a significant social economic burden on both developing and developed countries.

High myopia is significantly associated with morphological changes within the retina [6–8]. It increases the risk of pathologic myopia leading to retinal atrophy, lattice degeneration, lacquer cracks, choroidal neovascularization, and retinal detachment [9]. In Japan, myopic maculopathy was reported to be the primary cause of legal blindness and the third most prevalent cause of poor vision [10]. In Western populations, myopic maculopathy is also a significant cause of legal blindness [11].

OCTA is a technique that measure light reflectance of moving red cells to visualize the retinal vasculature and microcirculation. It is quick, non-invasive and provides quantitative, blood flow and structural information [12]. We attempted to utilize OCTA to investigate differences in macular vascular density in non-pathologic HM compared to non-high myopia (NHM) patients. It is hypothesized that with increasing myopia, there is lower vascular density within the macula, reducing perfusion and predisposing to pathologic myopia. Establishing differences in patients without evidence of eye disease is important aspect to understanding the natural history and etiology of pathologic myopia.

## Methods

### Participants

This is a prospective, cross-sectional study. Seventy eyes with myopia were recruited consecutively over the period of December 2014 to December 2020 in the Department of Ophthalmology in Peking Union Medical College Hospital, Chinese Academy of Medical Sciences. Thirty-five eyes with HM and 35 eyes with NHM were recruited. HM was defined as a spherical equivalence (SE) less than − 6.00D, and NHM defined as a SE greater than or equal to − 6.00D. Only the OCTA index of the right eyes of the participants were recorded. Exclusion criteria included any history of other any ocular diseases, systemic diseases that may affect the ocular circulation and previous intraocular surgery or ocular injury. Case files and OCTA images were reviewed and discussed by two expert ophthalmologists. Exclusion was based on mutual agreement. This study had ethical approval from the institutional ethics committee of the Chinese Academy of Medical Sciences, Peking Union Medical College Hospital, and all data collection were performed in accordance with relevant regulations and guidelines.

### Optical coherence tomography angiography

OCTA scans were obtained using OCT (Optovue, Fremont, California, USA) Angio-Retina mode (3 × 3 mm). Split-spectrum amplitude decorrelation angiography (SSADA) was utilized as the signal process algorithm. This has been described in detail in previous literature. Vascular density (VD) is the percentage of the sample area occupied by vessel lumens. OCTA images excluded from this study include those with poor signal strength (< 40), images with severe artefacts, images which demonstrates epiretinal membrane, foveoschisis, macular holes, choroidal neovascularization and retinal detachments. A 3x3mm scanning area of the macula was acquired and divided into the superficial, deep and choroidal layers. Each of the layers were automatically segmented by the software. Results were analyzed with the Optovue software. VD of the overall macula was calculated, and each region was calculated separately (fovea, parafovea, superior-hemi and inferior-hemi).

### Statistical analysis

SPSS software package (SPSS 12.0) was utilized to perform statistical analysis. The mean and standard deviation of the main parameters were calculated. The VD were compared between the two groups. A *p*-value calculated using the T-test, and a level of < 0.05 was considered statistically significant.

## Results

### Demographics

Seventy right eyes (32 men and 38 women) were included in this study. The NHM group (*n* = 35) contained 18 women and 17 men with a mean age of 27.3 ± 4.5 years. The HM group (*n* = 35) contained 20 women and 15 men with a mean age of 25.6 ± 5.4 years. There were no significant differences in gender, age, K1, K2, Avek and CCT between the two groups (Table 1). Forty-one patients were excluded from the study.

**Table 1:** Demographic and Corneal Baseline Data

	NHM	HM	*p*
Age (mean)	27.3	25.6	0.811
Male	17	15	0.181
Female	18	20	
SE (Diopters)	−3.46	−7.57	< 0.01
K1 (D)	43.45 ± 16.8	44.07 ± 14.2	0.090
K2 (D)	42.53 ± 14.9	42.71 ± 13.0	0.614
KMean (D)	42.91 ± 15.0	43.44 ± 12.9	0.117
CCT	536.94 ± 33.96	543.82 ± 33.32	0.458

*SE* Spherical Equivalence, *KMean* K mean, *CCT* central corneal thickness

### Macular density

The superficial retinal layer showed an overall decrease in VFD in the HM group (Table 2). The overall macular VD were 51.27 ± 3.74 and 48.07 ± 5.69 in the NHM and HM groups, respectively (*p* < 0.05). The multiple sectorial comparisons showed that there were significant reductions in the VFD in the HM group in the parafovea (52.58 ± 5.78 vs 49.40 ± 6.43, *p* < 0.05), superior-hemi (53.38 ± 4.03 vs 49.78 ± 6.84, *p* < 0.05) and inferior-hemi regions (53.49 ± 4.61 vs 49.05 ± 6.41, *p* < 0.05), but not in the fovea region (28.75 ± 5.22 vs 28.56 ± 6.55, *p* > 0.05).

**Table 2:** Macular Vascular Density in Superficial Retinal Layer of NHM and HM patients

	NHM (mm^**2**^ ± SD)	HM (mm^**2**^ ± SD)	***p***
Overall	51.27 ± 3.74	48.07 ± 5.69	0.017
Fovea	28.75 ± 5.22	28.56 ± 6.55	0.890
Parafovea	52.58 ± 5.78	49.40 ± 6.43	0.043
Superior-Hemi Region	53.38 ± 4.03	49.78 ± 6.84	0.019
Inferior-Hemi Region	53.49 ± 4.61	49.05 ± 6.41	0.005

The deep retinal layer showed also an overall decrease in macular VFD in the HM group (Table 3). The overall macular VFD were 58.69 ± 2.46 and 56.90 ± 4.08 in the NHM and the HM groups, respectively (*p* < 0.05). In the individual sectorial comparisons, only the superior-hemi region showed a reduction in VFD in the HM group (61.97 ± 2.68 vs 60.08 ± 3.98, *p* < 0.05). There were no significant differences in the in the fovea (25.24 ± 6.04 vs 24.98 ± 6.97), parafovea (62.44 ± 2.30 vs 61.10 ± 3.55) and inferior-hemi region (62.90 ± 2.43 vs 62.11 ± 3.50) (*p* > 0.05) (Table 3).

**Table 3:** Macular Vascular Density in the Deep Retinal Layer of NHM and HM patients

	NHM (mm^**2**^ ± SD)	HM (mm^**2**^ ± SD)	***p***
Overall	58.69 ± 2.46	56.90 ± 4.08	0.041
Fovea	25.24 ± 6.04	24.98 ± 6.97	0.872
Parafovea	62.44 ± 2.30	61.10 ± 3.55	0.074
Superior-Hemi Region	61.97 ± 2.68	60.08 ± 3.98	0.029
Inferior-Hemi Region	62.90 ± 2.43	62.11 ± 3.50	0.270

The choriocapillaris showed no significant differences in the HM and NHM groups overall and in each of the individual sectors (Table 4). The overall macular VFD were 65.50 ± 1.62 and 64.69 ± 2.29 in the HM and NHM groups respectively (*p* > 0.05). There was no significant differences between the HM and NHM patients in the fovea (65.11 ± 3.12 vs 63.48 ± 5.48), parafovea (65.39 ± 1.65 vs 64.56 ± 2.22), superior-hemi region(65.31 ± 1.65 vs 64.24 ± 3.15) and inferior-hemi region (65.49 ± 1.76 vs 64.86 ± 2.57) (*p* > 0.05).

**Table 4:** Macular Vascular Density in the Choriocapillaris of NHM and HM Patients

	NHM (mm^**2**^ ± SD)	HM (mm^**2**^ ± SD)	***p***
Overall	65.50 ± 1.62	64.69 ± 2.29	0.137
Fovea	65.11 ± 3.12	63.48 ± 5.48	0.138
Parafovea	65.39 ± 1.65	64.56 ± 2.22	0.119
Superior-Hemi Region	65.31 ± 1.65	64.24 ± 3.15	0.108
Inferior-Hemi Region	65.49 ± 1.76	64.86 ± 2.57	0.284

## Discussion

High myopia is well-known to be associated with significant histopathological changes of the retina [7] and can lead to myopic maculopathy. Various degenerative changes can become evident in the posterior segment. In many developed countries, myopic maculopathy remains one of the key causes of visual impairment [13].

Although the etiology of myopic maculopathy is still elusive, previous literature has suggested that increased radius of the eyeball in HM eyes lead to reduced blood circulation [14–16]. There is evidence that reduced choroidal blood flow over a prolonged period of time leads to release of vascular endothelial growth factor (VEGF) and consequent myopic choroidal neovascularization (CNV) [17]. Myopic CNV was shown to have a prevalence rate of 10-11% over the period of 12 years in one study [18]. The pathogenesis of myopic maculopathy may stem from a similar pathophysiological pathway.

Previous studies of retinal vasculature in HM eyes have focused on the use of time domain OCTs (TD-OCT) and Colour Doppler Imaging (CDIs). Axial length has been correlated with regional variations of retinal thickness [19] and negatively correlated with choroidal or retinal blood flow. Such studies have had difficulty visualizing the microvasculature, and experienced poor differentiation of static tissue to vasculature. Other techniques which have being utilized to investigate retinal blood flow include fluorescein angiography (FA). The utility of FA in HM is low due to its invasive and qualitative nature. Any potential side effects are further avoided in OCTA.

OCTA allows detailed, three-dimensional study of vasculature down to the capillary level. It utilizes the intrinsic motion of blood cells present in the vascular networks, offering a non-invasive and rapid test without the need for intravenous contrast [20].

In our study, OCTA was utilized to compare the macular VD of patients with NHM and HM without pathologic myopia. The SE of − 6.00D was utilized to differentiate between the two groups. Our study demonstrates that HM is associated with a reduced macular VD. This finding supports the hypothesis that reduced macula perfusion leads to pathologic myopia. Further studies are required to explore the exact pathophysiological mechanism for mean decrease in macula vascular density.

Multiple studies have demonstrated decreasing macular VD being associated with increasing myopia [21, 22]. Some studies have demonstrated only differences in certain regions and layers [23, 24], whilst others have not demonstrated any differences between high myopic and non-high myopic patients [25–27]. This difference in the literature could be attributable to selection of population. Studies which demonstrated non-significant difference between VD of myopic and high-myopic patients had relatively narrow age ranges of patients [25]. Although the pathogenesis of myopic maculopathy is still relatively unknown, prognosis of patients with myopic maculopathy worsens with age [28]. With increasing age, a greater reduction of macular VD occurs. In sample sizes with younger patients, changes in VD may not be reflected. Future studies are needed with larger data-sets, with greater time-frames to allow for accurate complex multi-variable analysis. A systematic synopsis of the current published studies would be highly important.

Limitations to our study includes the lack of regression analysis to examine for other factors such as axial length which could contribute to the reported differences in macula vascular density. Although there has not been formal power analysis undertaken, our study is of similar size to previous published investigations [24].

In conclusion, our study has demonstrated negative correlations between high myopia and both the superficial and deep retinal vascular flow density readings as attained by OCTA.

## Data Availability

Data supporting the results reported in the article are not public but can be accessed after communicating with the corresponding author.
